# Ether Spray as an Anæsthetie

**Published:** 1876-02

**Authors:** 


*The Ether Spray as an Anœsthetic.*—Mr. Thomas Moore, M. R. C. S., describes the following case of excision by the scissors under ether spray in the *London Lancet*:—

A maiden lady, aged sixty-four, came, on September 9th, from a town fifty miles distant, to place herself under my care. She was suffering from a very hard scirrhus tumor of the left breast, rather larger than a turkey's egg, which she had first noticed eight months before, when it was as large as a walnut.

On the 14th of September the operation was performed as follows:—First a spray of common ether was directed on to the breast for five minutes; then two sprays of anæsthetic ether were directed—the one by Mr. Cross, and the other by our assistant, Mr. Bayfield—on either side of the tumor, for five more minutes, until the whole breast was frozen quite hard. I then, after making an angular cut in the skin at the outer margin of the tumour, carried the lower blade of the cutting-scissors deeply down through the breast until it appeared to rest upon the pectoral muscle. Then, keeping this blade as deep as possible, I easily cut the lower flap, about an inch from the tumor, with three or four strokes of the scissors. The upper flap was cut as easily. I next thrust the fingers and thumb of my left hand deep into the wound, and, grasping the tumor firmly, raised it as far as possible from the pectoral muscle. It was then easy to detach it with a few strokes of a pair of tooth-edged scissors. The operation so far was completed in less than three minutes from the time of the commencement of the first incision, and was attended with very little loss of blood. When the spray was withdrawn there was some hemorrhage from three small vessels in the tissues which had been cut by the sharp-edged scissors, two of which were treated by torsion, and the other tied with silk, the ends of the ligature being cut off short. The edges of the wound were brought together by six sutures, and each of the twelve spots through which the needle was passed was previously separately frozen by the spray.

My patient, who declared beforehand that she was extremely sensitive to pain, gave very little indication of having felt any during the operation, and said afterward that "it hurt her, but not a great deal." That her estimate of her extreme sensitiveness was correct, (and indirectly the success of the anæsthetic) was proved by the fact that, during the introduction of one of the sutures through a part of the skin which had been accidentally insuffiiently frozen, she cried out loudly, and declared that this "hurt her worse than all the rest put together."

The wound, which was dressed with oiled lint and cotton-wool, united by first intention, except in a spot where I had made a slight notch at the junction of two cuts of the scissors, and at the place of the first incision ; but there never was more than just enough discharge to moisten the dressing, and that was entirely dried up by the sixteenth day. The patient was confined to her bed for one day, and to her bedroom and an adjoining sitting-room for a week.

The scissors used for making the first incisions were ordinary

"elbow-scissors," with the blades inclined to the handles at about a third of a right angle; those for making the deeper incisions were tooth-edged and slightly curved "on the flat of the blade."

---

### MONTHLY SUMMARY.

